# Unraveling the Atomic Mechanism of the Crystalline Phase‐Dependent Structural Features and Special Spectral Design of α‐, β‐, and Ɛ‐Ga₂O₃

**DOI:** 10.1002/advs.202508207

**Published:** 2025-06-23

**Authors:** Xinqing Han, Yong Liu, Yang Li, Miguel L. Crespillo, Eva Zarkadoula, Wenxiang Mu, Peng Liu

**Affiliations:** ^1^ Shandong Provincial Key Laboratory of Nuclear Science Nuclear Energy Technology and Comprehensive Utilization School of Nuclear Science Energy and Power Engineering Shandong University Jinan Shandong 250061 P. R. China; ^2^ State Key Laboratory of Crystal Materials and Institute of Novel Semiconductor Shandong University Jinan Shandong 250100 P. R. China; ^3^ Department of Nuclear Engineering University of Tennessee Knoxville TN 37996 USA; ^4^ Center for Nanophase Materials Sciences Oak Ridge National Laboratory Oak Ridge TN 37831 USA; ^5^ Institute of Frontier and Interdisciplinary Science and Key Laboratory of Particle Physics and Particle Irradiation Shandong University Qingdao 266237 P. R. China

**Keywords:** electronic state configuration, intense electronic excitation, spectral decomposition, structural phase transition, thermodynamic response

## Abstract

Atomic‐scale phase transformations profoundly influence the functional properties of Ga₂O₃ polymorphs. By combining irradiation experiments with microstructure characterization and theoretical approaches, phase‐specific energy‐dissipation pathways in α‐, β‐, and ε‐Ga₂O₃ are uncovered and strategies for targeted property design are outlined. Competing antiphase boundaries (APBs) and twin domain boundaries (TDBs) promote irreversible α→ε interconversion through domain fragmentation. In β‐Ga₂O₃, defect‐induced stress gradients drive two distinct local transformations: surface Ga‐aggregated β→δ that stabilizes transient states, and latent‐track‐confined β→κ phase transition with recoverable distortions via cation reordering. Under electronic excitation, β‐Ga₂O₃ forms nanohillocks via robust GaO₆ octahedra (high density/strong Ga─O bonds), while α/ε‐Ga₂O₃ generates nanopores from tetrahedral Ga looseness (low bonding energy), highlighting phase‐dependent surface dynamics shaped by atomic packing and bonding anisotropy. Defect‐regulated recombination suppresses visible photoluminescence in α/β‐Ga₂O₃, whereas in ε‐Ga₂O₃ bandgap narrowing of ΔE: 0.30 eV is observed, enhancing emission. Linking phase‐dependent defect‐carrier interactions and metastable‐phase engineering in Ga₂O₃ enables property optimization for power‐electronics and optoelectronics devices.

## Graphical abstract

Understanding atomic‐scale phase transformations and their spectral responses is key to tailoring semiconductor properties for specific applications. Phase‐dependent thermodynamic responses in α‐, β‐, and ε‐Ga₂O₃ reveal distinct lattice energy dissipation pathways. Critical melting energies (0.22–0.34 eV atom^−1^) correlate with specific electron‐phonon coupling coefficients, driving preferential α/ε interconversion via domain fragmentation, while β‐Ga₂O₃ undergoes localized β→κ/δ transitions.

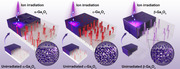

## 1 Introduction

In contemporary materials science, advancements are propelled by innovative technologies and breakthroughs focused on enhancing the properties of existing materials and substituting them with more efficient and appropriate alternatives. Semiconductor compounds exemplify this trend, with advancements in cutting‐edge power devices driving the development of next‐generation high‐power electronics and enabling diverse applications^[^ ^1^, ^2^, ^3^, ^4^, ^5^ ^]^ including biomedical imaging technologies,^[^ ^6^, ^7^, ^8^, ^9^ ^]^ luminescent photovoltaic concentrators,^[^ ^10^, ^11^, ^12^, ^13^ ^]^ quantum‐confined nanostructure lasers,^[^ ^14^, ^15^, ^16^ ^]^ and high‐resolution emissive display platforms.^[^ ^17^, ^18^, ^19^ ^]^

While silicon carbide (SiC), a prototypical wide‐bandgap semiconductor (WBG, *E_g_* ≈3.3 eV), has emerged as the industry‐standard workhorse for modern power electronic systems, the scientific frontier is now shifting toward ultrawide‐bandgap(UWBG, *E_g_* > 4.5 eV) semiconductor technologies that exhibit paradigm‐shifting potential for extreme‐performance electronic devices operating in multi‐kilovolt (> 10 kV) and high‐temperature (> 500 °C) regimes.^[^ ^20^, ^21^, ^22^, ^23^, ^24^, ^25^ ^]^ This material family encompasses several promising candidates including aluminum nitride (AlN, *E_g_* ≈6.2 eV) with superior thermal conductivity, cubic boron nitride (c‐BN, *E_g_* ≈6.4 eV) exhibiting exceptional hardness, and diamond (*E_g_* ≈5.5 eV) possessing ultrahigh carrier mobility.^[^ ^26^, ^27^, ^28^, ^29^ ^]^

The polymorphic versatility of Ga₂O₃, featuring five distinct phases (α, β, γ, δ, and ε), enables unprecedented tunability in wide‐bandgap semiconductor design through crystal engineering.^[^ ^30^, ^31^, ^32^, ^33^ ^]^ While β‐Ga₂O₃ (monoclinic, *E_g_*: 4.8–4.9 eV) dominates power electronics with superior Baliga's figure of merit (BFOM > 3000) and thermodynamic stability, metastable phases offer complementary functionalities: α‐phase (corundum, 5.3 eV) enhances deep‐UV optoelectronics, ε/κ‐phase (hexagonal) demonstrates exceptional polarization switching (8.0 MV cm^−1^ breakdown field) for neuromorphic computing, and γ‐phase (defect‐spinel) shows enhanced surface reactivity for gas sensing. This polymorphic engineering approach allows strategic optimization of electronic parameters, with bandgap energies spanning 4.7–5.3 eV across phases and carrier mobility varying by two orders of magnitude depending on crystal symmetry, establishing Ga₂O₃ as a platform for next‐generation electronics where phase coexistence enables the simultaneous achievement of high‐power switching, optoelectronic functionality, and thermal control within monolithic systems.^[^ ^34^, ^35^, ^36^, ^37^, ^38^, ^39^, ^40^, ^41^, ^42^ ^]^

Ion beams in the semiconductor domain serve as a state‐of‐the‐art tool with multifaceted utility in fundamental research and industrial applications, enabling the breach of traditional equilibrium methods to surmount the limitations of impurity solubility in Ga_2_O_3_ semiconductors, and employing strategies of defect engineering‐dominated by intense electronic excitation and nuclear‐nuclear collisions.^[^ ^43^, ^44^, ^45^, ^46^, ^47^ ^]^ From the point of view of fundamental research, the various vacancies or gaps created to act as a potential disorder, affecting in principle the electrical transport properties (e.g., carrier mobility) and thus highlighting the contribution of Anderson localization, while the introduced defects lead to new energy levels located in the bandgap strongly affecting the transport behavior, mainly by modulating the carrier concentration.^[^ ^48^, ^49^, ^50^, ^51^, ^52^ ^]^ Unlike metals, where electrical and thermal transport properties are linearly related through the Wiedemann‐Franz law due to the shared role of free electrons as carriers, Ga_2_O_3_ semiconductors are primarily governed by electrons (or holes) for electrical conduction and phonons for thermal transport, exhibiting unique properties that can be precisely engineered by irradiation modifying composition and crystal structure.^[^ ^53^, ^54^ ^]^ Therefore, multi‐objective optimization that simultaneously considers both electron and phonon properties is ultimately critical to realizing the Ga_2_O_3_ semiconductor by design for high‐power electronics, deep‐ultraviolet photodetectors, and radiation‐hardened materials.^[^ ^55^, ^56^ ^]^

In the present research on intense electronic excitation in Ga₂O₃, the atomic mechanism of phase‐dependent structural dynamics and spectral features of α‐, β‐, and ɛ‐Ga₂O₃ are systematically explored from the critical atomic‐scale structure characteristics and thermodynamic behavior in governing phase transformation. Phase‐dependent thermodynamic responses revealed energy dissipation‐induced behaviors and triggered differentiated structure features located in different microregions, connected with phase‐specific bandgap narrowing and recombination pathways, bridging atomic defect mechanisms to macroscopic performance, and explaining the contrasting photoluminescence yields between and α‐, β‐, and ε‐phases. Phase‐transition engineering of Ga₂O₃ polymorphs enables the on‐demand modulation of ultrawide‐bandgap (UWBG) functionality, where controlled phase transformations directly reconfigure the critical electronic structure. Consequently, a systematic investigation of irradiation‐induced phenomena among the α‐, β‐, and ε‐Ga₂O₃ phases is essential to determine the requisite conditions for initiating the α‐ to ε‐ and β‐ to δ/κ‐phase polymorphic transformations, which are of significant importance for the design and development of solar‐blind photodetectors and programmable quantum emitters.

## 2 Results and Discussion

### 2.1 Irradiation‐Induced Distortion Anisotropy and Phase Structure Interconversion

**Figure 1:**
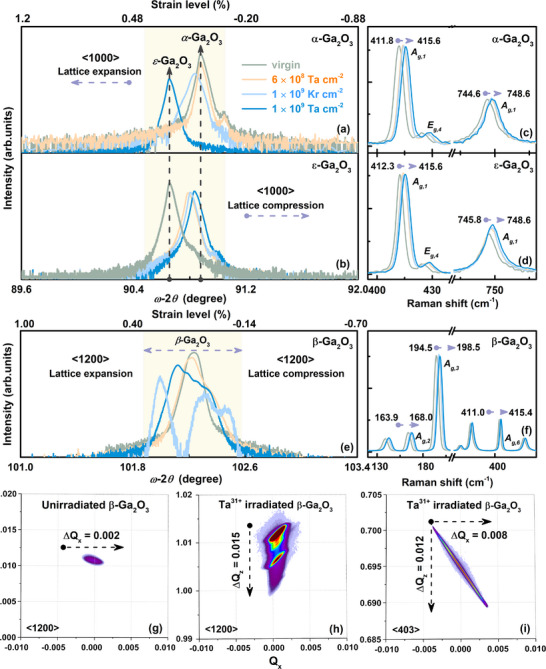
Material characterization and stress analysis of α, ε, and β‐Ga_2_O_3_. (a,b,e) HRXRD *ω‐*2*θ* scans patterns of pristine and Kr^17+^ and Ta^31+^ irradiated α, ε, and β‐Ga_2_O_3_ around ⟨1000⟩ and ⟨1200⟩ reflection, with strain level obtained using Bragg equation. (c,d,f) Experimental Raman scattering spectra of α, ε, and β‐Ga_2_O_3_ excited at λ = 532.0 nm at room temperature (RT). (g–i) RSM around the asymmetrical node (⟨1200⟩ and ⟨403⟩) of a structure consisting of a distortion layer and original undamaged lattice configurations of β‐Ga_2_O_3_, involving the evolution of the in‐plane (Q_x_) and out‐of‐plane (Q_z_) strain.

The essential characterization of X‐ray diffraction (XRD) combined with reciprocal space mappings (RSM) are applied to structurally analyze the residual strain and lattice relaxation of α, ε, and β‐Ga_2_O_3_ crystallographic evolution in both in‐plane and out‐of‐plane directions, utilizing varying Kr^17+^ and Ta^31+^ irradiation with fluence from 6.0 × 10^8^ to 1.0 × 10^9^ cm^−2^. The kinematics features around ⟨1000⟩ and ⟨1200⟩ reflection are summarized as follows: (i) The zero‐order peak, caused by the Bragg reflections from the lattice planes in the analogical well and the barrier layers. (ii) Diffraction peaks moving toward low angles correspond to lattice expansion in α‐Ga_2_O_3_, while diffraction peaks moving toward high angles correspond to lattice contraction in ε‐Ga_2_O_3_, and eventually converge to a uniform position, reflecting the trend of the two crystalline phases to interconvert response to specific electronic excitation (**Figure** **1**a,b). (iii) A dominant orientation is continuously detected for the β‐phase, despite a lower intensity and extended spreading broadly on both sides of the peaks concerning pristine β‐Ga_2_O_3_ (Figure 1e).

Polarized Raman spectra, corresponding to characteristic peaks expected from the polarization selection rules,^[^ ^57^ ^]^ exhibit varying degrees of blue shift in Figure 1c,d,f. Particularly, the *A_g_, _1_* and *E_g_, _1_* present a consistent shifting trend toward higher frequencies in α and ε‐Ga_2_O_3_, eventually reaching 415.6 and 748.6 cm^−1^, distinguishing the Raman peak position in β‐Ga_2_O_3_, reflecting libration and translation of the Ga_I_O_4_ and Ga_II_O_6_, and stretching and bending of Ga_I_(O_I_O_III_) bonds in non‐uniformity of crystalline orientation and consistent vibrational behaviors between α and ε‐Ga_2_O_3_. Focusing on the anisotropic behavior of lattice distortions in the β‐phase, the weakened displacement field around the structural defects results in diffuse scattering, characterized by a relatively nonuniform, anisotropic intensity distribution, primarily from the random elastic deformation of the host defects lattice. Compared with original diffraction peaks in pristine β‐Ga_2_O_3_ (Figure 1g), an elongated diffracted signal Q_x_ spreads along the ⟨0001⟩ direction, parallel to the surface, appearing in irradiated β‐Ga_2_O_3_, and the Q_z_ component observed in the ⟨403⟩ crystal orientation, confirming that elastic strain due to lattice distortion co‐occurs in both directions and the comparison of changes (Q_x_ = 0.008 < Q_z_ = 0.012) further reveals the anisotropic feature of the distortion (Figure 1h,i).

### 2.2 Thermodynamic Response Toward α‐, β‐, and ɛ‐Ga₂O₃ Under Electronic Excitation

**Figure 2:**
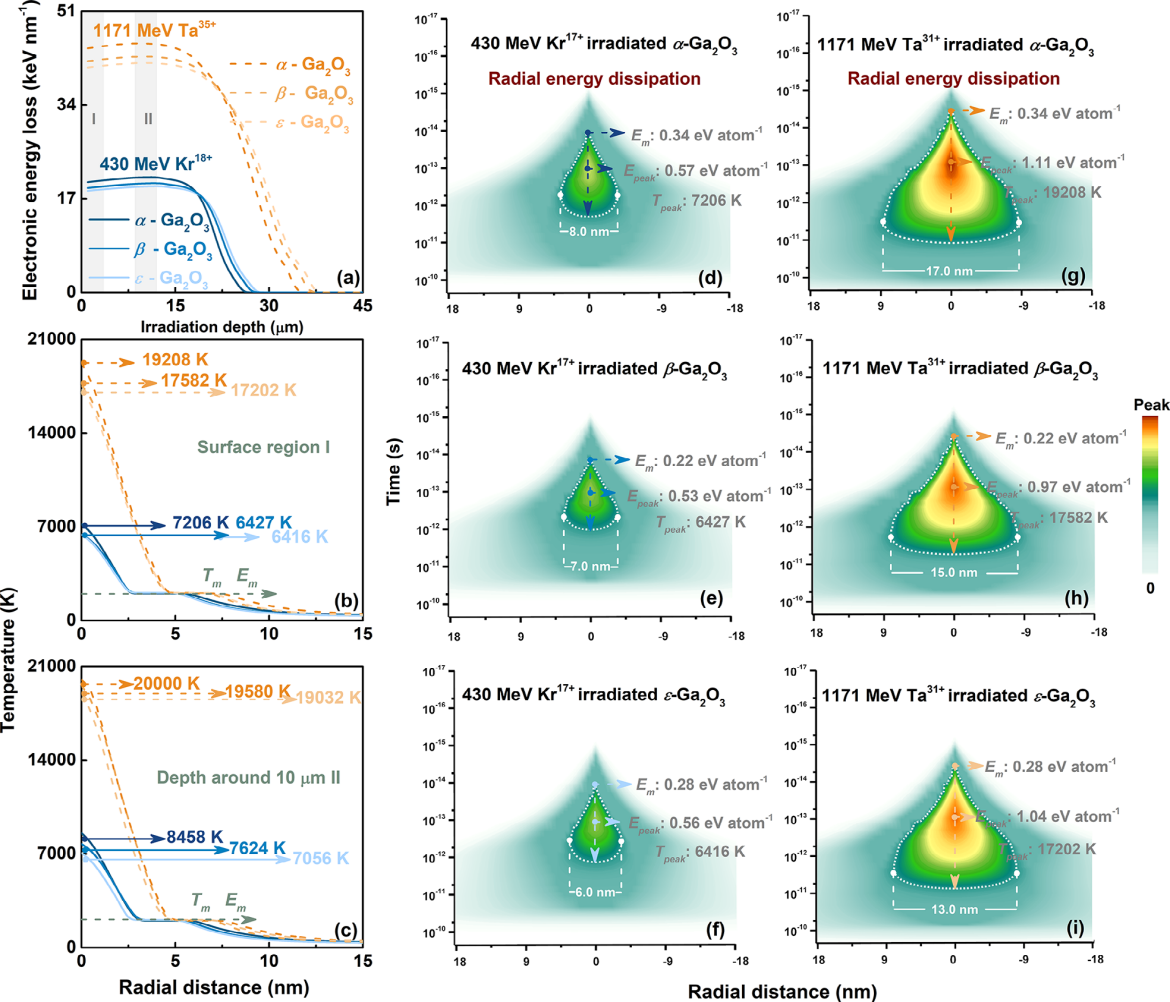
Energy deposition driven by thermal spike response in the two‐temperature model. (a–c) SRIM‐simulated *E_ele_* depth profile and electronic excitation induced energy deposition to atomic subsystems in the surface and 10 µm depth damage layers, and (d–i) surface spatiotemporal evolution of radial energy distribution of α, β, and ε‐Ga_2_O_3_ under Kr^17+^ and Ta^31+^ irradiation, labeled with different melting thresholds *E_m_*.

Responding to intense electronic excitation, high‐density kinetic and potential energies were transferred to the α, β, and ε‐Ga_2_O_3_ lattice, triggering thermodynamic behavior dependent on varying crystalline phase structure, facilitating energy transfer and deposition to the lattice followed by dissipation during the relaxation process. Considering electronic energy loss *E_ele_* from 40.0 to 44.0 keV nm^−1^ and potential energy *E_p_* of 42.8 keV nm^−1^ (calculated by Coulomb Potential Energy Formula^[^ ^58^ ^]^) under Ta^31+^ irradiation and *E_ele_* from 18.5 to 20.0 keV nm^−1^ and *E_p_* of 16.9 keV nm^−1^ under Kr^17+^ irradiation simulated by SRIM (**Figure** **2**a),^[^ ^59^, ^60^ ^]^ the radial energy distribution calculated by iTS model near‐surface and interior layer (≈10 µm), exceeding the melting point *T_m_*, were clearly contrasted in Figure 2b,c.

Focusing on thermodynamic systems, the melting criteria of energy *E_m_* in terms of thermal spike response for melting phase formation were calculated to be 0.34, 0.22, and 0.28 eV atom^−1^ for α, β, and ε‐Ga_2_O_3_ based on the expression $E_{m}=\int_{0}^{T_{m}}\;C_{a}\left(T_{a}\right)dT_{a}$, where *T_m_* was set to 2073, 2013, and 2073 K, respectively.^[^ ^61^, ^62^, ^63^, ^64^ ^]^ Subsequently, different radial energy dissipation profiles were comparatively analyzed under an identical gradient scale of the deposition energy: (i) under the Kr^17+^ irradiation (Figure 2d–f), the *E_peak_* far exceeds the *E_m_* in α‐Ga_2_O_3_, reaching 0.57 eV atom^−1^ (≈7206 K), corresponding to a melting diameter of ≈8.0 nm, higher than the *E_peak_* over *E_m_* to 0.53 eV atom^−1^ (≈6427 K) and 0.56 eV atom^−1^ (≈6416 K), accompanied by melting diameters of ≈7.0 nm and ≈6.0 nm in β and ε‐Ga_2_O_3_. (ii) Similarly, under the Ta^31+^ irradiation (Figure 2g–i), the deposition energy at track center increases nonlinearly with enhanced electronic excitation to 1.11 eV atom^−1^ (≈19 208 K), far exceeding the *E_peak_* of 0.97 eV atom^−1^ (≈17 582 K) and 1.04 eV atom^−1^ (≈17 202 K) in β and ε‐Ga_2_O_3_, corresponding melting diameter are ≈17.0 nm ≈15.0 nm, and ≈13.0 nm, respectively. By comparison, the *E_m_*, acting as a barrier to lattice melting and obstructing damage formation, are in the proper sequence to 0.34, 0.22, and 0.28 eV atom^−1^, accompanied by decreased *E_peak_*, associated with phase‐dependent *e‐ph* coupling response in thermal spike simulations, as displayed in **Table** **1**.

**Table 1:** The i‐TS model calculation of electronic structural properties in α‐, β‐, and ε‐Ga_2_O_3_ systems.

Comparison of electronic structural properties of *α‐, β‐, ε‐*Ga_2_O_3_ systems along <100> orientations (*RT* ≈300 K)				
Sample	Orientation	The *e‐ph* coupling coefficient	The *e‐ph* means free path	Bandgap
		[10^18^ W m^−3^ K^−1^]	[nm]	[eV]
Ga_2_O_3_	α ⟨100⟩	5.66	4.2	5.29
	β ⟨100⟩	4.34	4.8	4.71
	ε ⟨100⟩	4.72	4.6	5.16

Furthermore, a larger intrinsic band gap generally correlates with a reduced mean free path that amplifies the scattering phase space, thereby intensifying interactions between electrons and lattice vibrations (e.g., via deformation potential coupling mechanisms), eventually enhancing thermal peak response between α, β, and ε‐Ga_2_O_3_. Meanwhile, the multiphase transformation is inclined to occur in α and ε‐Ga_2_O_3_ with a similar thermal response, reflecting the crucial impact of energy transfer in electronic and atomic systems, consistent with the mutual transformation of diffraction patterns (Figure 1a,b). Additionally, in the prediction of the final structure of α, β, and ε‐Ga_2_O_3_, damage recovery must be prioritized as a critical consideration, as it governs the dynamic evolution of structural integrity and defect annihilation mechanisms during post‐processing or operational conditions.

### 2.3 Structural Characteristics and Elemental Distribution Driven By Thermodynamics

**Figure 3:**
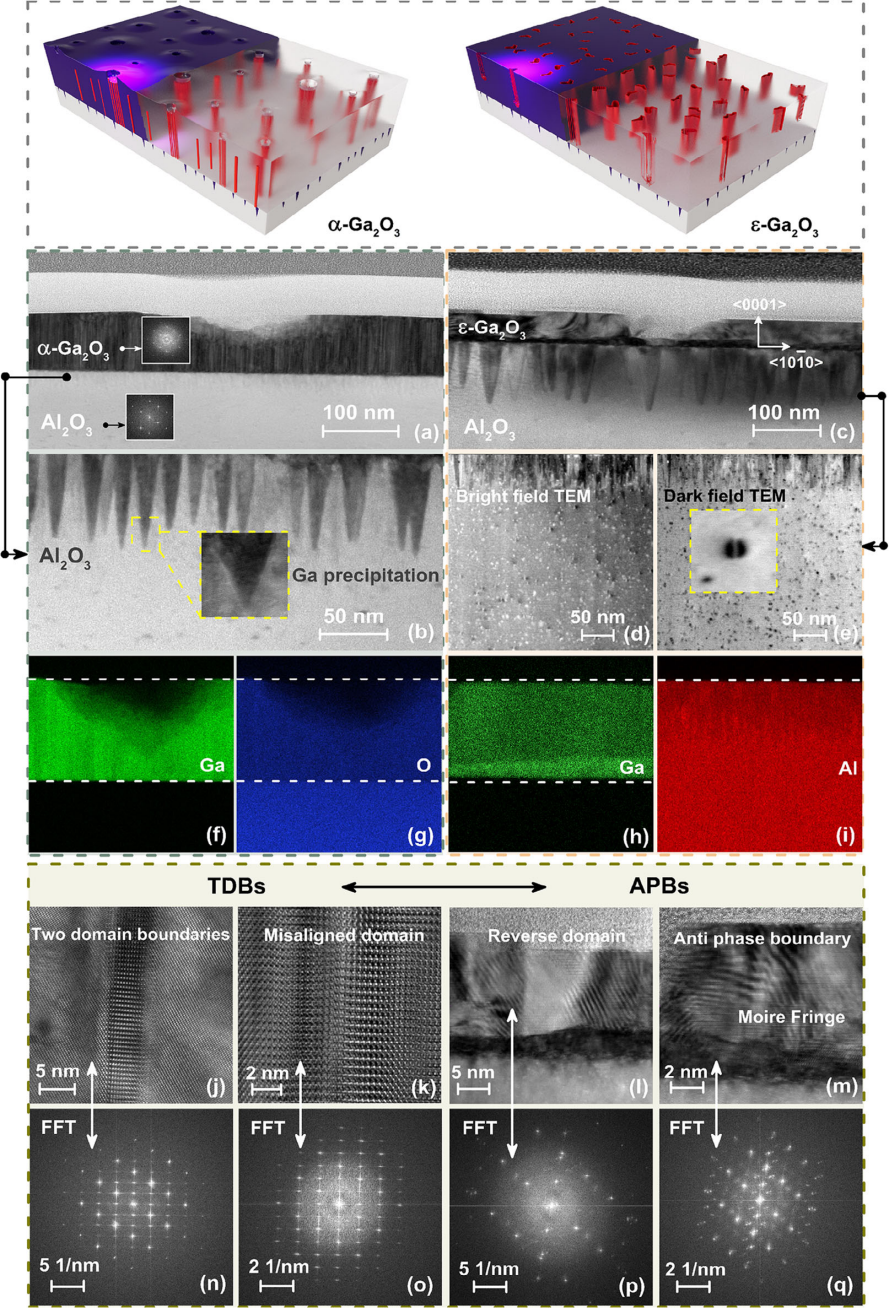
Representative crystalline nanostructure between α and ε‐Ga_2_O_3_. (a, c) High‐angle annular dark field‐scanning TEM (HAADF‐STEM) images observed from the <0001> axis out‐of‐plane, involving the top, middle, and interface regions, and (b, d, e) localized boundary magnification with element precipitation and neighboring matrix area. (f–i) Quantified EDX elemental distribution mappings of Ga, O, and Al from heteroepitaxial of manufactured α‐ and ε‐Ga_2_O_3_, matching the intended thickness and compositions in cross‐sectional view. (j‐m) High‐resolution structural comparison: domain boundary types with stacking fault and Moire fringe obtained from localized magnification dominate in α‐ and ε‐Ga_2_O_3_, respectively, (n–q) corresponding to Fourier‐transform (FFT) patterns dominated from the phase domain structure.

High‐resolution TEM characterization combined with quantified EDX elemental distribution mapping investigates structural components and defect evolution, further elucidating the relationship of phase transformation between α and ε‐Ga_2_O_3_ response to electronic excitation. The color‐assigned EDX elemental maps of gallium (Ga), oxygen (O), and aluminum (Al) exhibit a strong and sharp contrast across the film layer with a slightly curved termination at the sapphire substrate interface of α and ε‐Ga_2_O_3_. Compared to the original lattice structure in the unirradiated α‐Ga_2_O_3_ (see Figure S1a–d for details, Supporting Information), the crater on the surface and interior columnar domain structure (cross‐section of the columns), accompanied by Ga elemental deficiency, is revealed by amplitude and phase contrast, presenting columns of straight‐line APBs with a diameter of ≈5.0–7.0 nm (**Figure** **3**a, b, f, g).

Specifically, domain boundaries are proposed as favorable sites for the accumulation of point defects, thereby more electrons are diffracted when the domains are aligned with the correct crystal axes, resulting in a darker contrast than other regions following the Bragg reflection condition.^[^ ^65^ ^]^ The domains were delineated by planar defects, predominantly twin boundaries, whose structural characteristics correspond remarkably with the previously proposed idealized atomic model for the analogous orthorhombic alumina material, evidenced by FFT patterns in the illustrations (Figure 3a). Considering the migration of Ga driven by energy deposition‐induced precipitation tendency, a series of Ga nanopillars nearly arranged in the edge region from atomic‐scale resolution crystal structure features are proved to exist between the Ga_2_O_3_ and Al_2_O_3_ interface layer (Figure 3b), evidencing localized positions containing 0.13% of Ga aggregated in an Al_2_O_3_ matrix (Figure 3(f) and (g)).

Contrastingly, localized magnification of surface microregions reveals partial nanopore structures, with the surface layer consisting of irregularly shaped nanodomains, where the presence of TDBs and numerous bunches of APBs in each of the domains was established in ε‐Ga_2_O_3_ (Figure 3c). Distinguished with boundaries that possess coherent atomic structure without dangling bonds in irradiated α‐Ga_2_O_3_ (Figure 3j,k), a series of nanopores are built up. These presumably originate from numerous disoriented nanodomains (with a high density of the APB irregularities) in ε‐Ga_2_O_3_, with the appearance of numerous dark stripe‐like contrasts non‐uniformly distributed over the cross‐sectional layer (Figure 3l,m), with corresponding FFT patterns diffraction characteristics (Figure 3n–q). Combined with the strain‐induced mechanism, the translational shift between crystals on either side of TDBs introduces localized strain fields. These trigger periodic lattice variations and Moiré fringes at the interface (Figure 3l,m), where Ga segregation‐induced inhomogeneous boundary component distributions under bright and dark field patterns (Figure 3d,e), distinguished from the uniform boundary in unirradiated ε‐Ga_2_O_3_ (see Figure S1e–h, Supporting Information for details), were confirmed by EDX elemental maps (Figure 3h,i). The dominant APBs in ε‐Ga_2_O_3_ manifest either as isolated defects or as multi‐defect zones characterized by sequential atomic displacements, whereas the majority of TDBs exhibit incoherent structures and function as interfacial demarcations for the termination of APBs,^[^ ^66^ ^]^ with hexagonal lattice with regular coalescence at a rotation angle of 120° reflected in FFT patterns (Figure 3p,q).

In summary, the competitive interaction between APBs and TDBs emerges as the predominant factor governing the distribution of Ga_2_O_3_ constituents within the α‐ and ε‐phases, with analogous domain configurations and comparable elemental enrichment precipitation characteristics, driving inter‐conversion with each other. In α‐Ga₂O₃ (corundum structure, hexagonal symmetry), APBs arise from lattice translation symmetry breaking, and localized energy deposition promotes shear stress, enabling APBs to reconfigure into TDBs via atomic partial dislocation glide, minimizing strain energy. Conversely, weak interlayer bonding allows shear‐induced TDBs formation in ε‐Ga₂O₃ (orthorhombic/hexagonal layered structure), and irradiation disrupts layered stacking via electronic excitation, triggering APBs nucleation by disrupting cation ordering. The energy deposition‐induced fragmentation of TDBs into discrete APBs segments results in the generation of T‐like APB intersections in ε‐Ga_2_O_3_, compared with APBs‐dominated defect atomic arrangements in α‐Ga_2_O_3_, verifying the specific phase transformation between α‐ and ε‐Ga_2_O_3_. Structural stability aspects, electron beam insensitive properties stem from the unique anisotropic structural configuration of corundum‐structured α‐Ga₂O₃ [space group R‐3c] and ε‐Ga₂O₃ [P6₃mc], where the directional Ga─O bond network (bond lengths: 1.85–2.08 Å) creates an elevated energy barrier (> 3.5 eV) for defect migration across APB/TDB interfaces, enabling reliable nanoscale characterization of metastable Ga₂O₃ phases without beam‐induced artifacts.^[^ ^67^ ^]^

**Figure 4:**
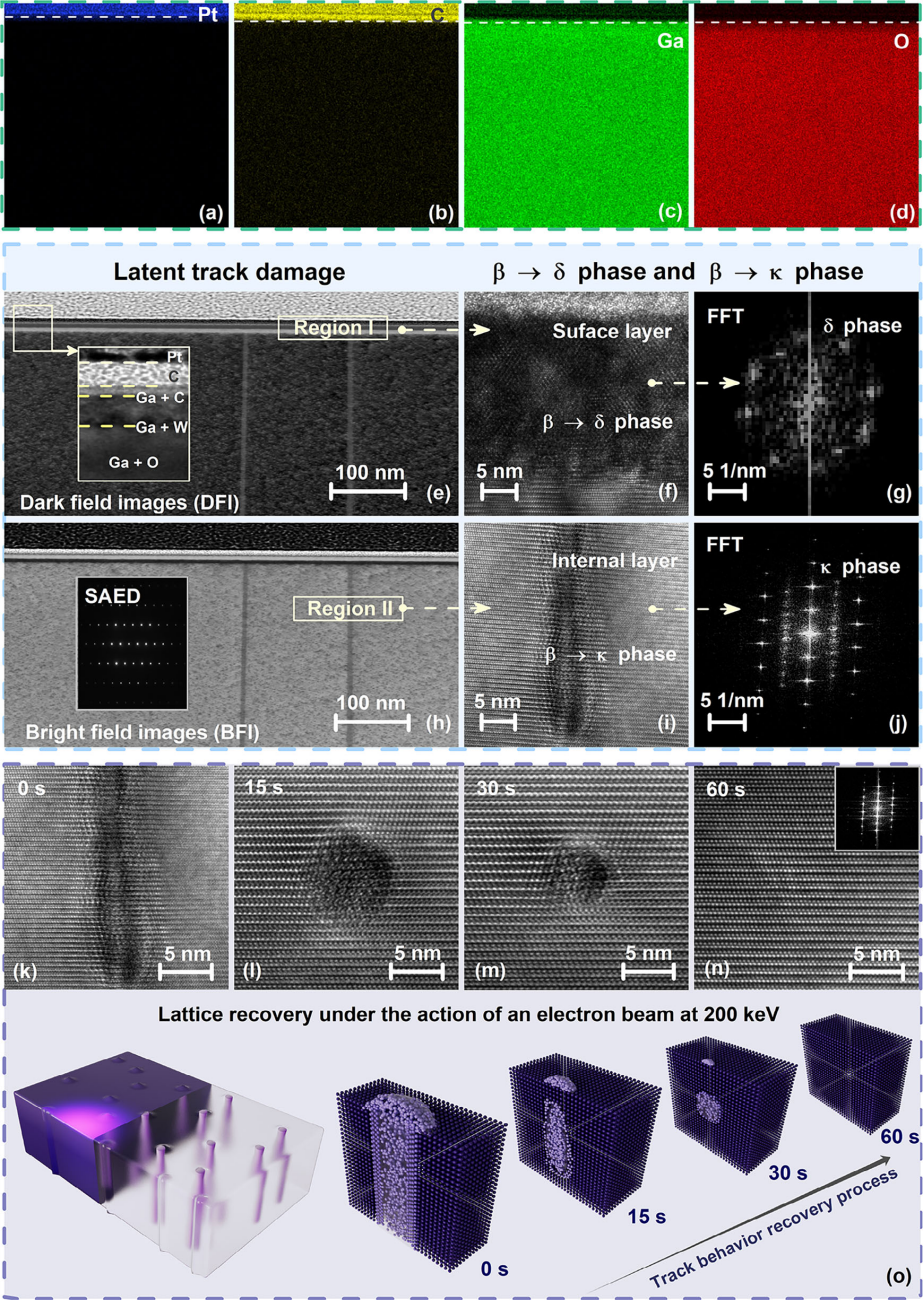
Representative nanostructure transformation of β‐Ga_2_O_3_. (a–d) Quantified EDX elemental distribution mappings of Pt, C, Ga, and O extract cross‐sectional β‐Ga_2_O_3_ sample from FIB preparation. (e, h) Irradiation‐induced phase transition behavior among latent tracks under dark/bright fields, associated with the acquired SAED, is dominated by the respective layer elements. The *κ*‐ and *δ‐*phase nucleation is distributed in the (f, g) surface and (i, j) interior microregion. (k–n) The evolutionary process of multiple crystal nuclei recovery to the β‐Ga_2_O_3_ responses to 200 keV electronic excitation. (o) Graphic representation of lattice recovery under the action of 200 keV electron beam.

The irradiation‐induced structural modifications and subsequent restoration processes at the atomic level, investigated employing advanced characterization techniques with spatial resolution, reveal fundamental insights into defect dynamics and lattice restoration processes in β‐Ga_2_O_3_. The structural evolution pathways in Ga_2_O_3_ polymorphs demonstrate distinct characteristics, as evidenced by their unique phase transformation behaviors. The color‐assigned EDX spectroscopy elemental maps, depicting the distribution of platinum (Pt), carbon (C), gallium (Ga), and oxygen (O) in **Figure** **4**a–d, reveal no significant elemental variations compared to the trajectory damage region, indicating minimal compositional alterations within the analyzed area. In the case of α‐ and ε‐Ga_2_O_3_, the structural evolution is predominantly governed by domain structure, and the APBs nucleation starts to appear at the TDBs, as illustrated in Figure 3j–m.

Conversely, the monoclinic β‐Ga_2_O_3_ system exhibits localized phase transformations, specifically β → κ and β → δ, which have been unambiguously confirmed through bright‐field and dark‐field TEM imaging (Figure 4e,h), driving significant hierarchical structural components in the illustration. The spatially segregated phase transformations, characterized by the β → δ transition predominantly occurring in surface microregions, accompanied by significant Ga aggregation, and the β → κ transition localized within latent track damage regions, are attributed to the specific configurations of localized defects and their corresponding stress fields inherent to the monoclinic β‐Ga_2_O_3_ system, underscoring the critical role of defect‐mediated mechanisms in governing the structural evolution of Ga_2_O_3_ polymorphs. In the transition region, the rapid decrease in bond breakage and formation, coupled with the migration of interstitial atoms to the outer layers, leads to a gradual density increase that matches that of the unirradiated regions, ultimately disrupting the element composition and facilitating a stable transition from β‐ to κ‐phase (Figure 4f). Toward the end of the transformation, when approaching the interface and external surface, heat is dissipated more promptly, and negative pressure induced by vacancy thermal migration is sufficient for the formation of the δ‐phase (Figure 4i), which contains high‐density antiphase boundaries, verified by FFT patterns of the diamond‐type structure Ga_2_O_3_ polymorph (Figure 4g,j).

Returning to the underlying recovery mechanism, owing to localized electronic excitations that lower energy barriers for the rearrangement of interfacial atoms, the κ‐phase, characterized by a corundum‐like structure with lower thermodynamic stability compared to the monoclinic β‐phase, exhibits higher susceptibility to structural perturbations (e.g., vacancies, interstitials, or antisite defects) acting as nucleation sites for phase transformation, lowering the energy barrier for atomic rearrangement toward the β‐phase configuration. Eventually, considering the activation energy for e‐beam (200 keV) enhanced unstable phase transitions, the κ‐phase dominates the reduction of the diameter of the radial latent track and the shortening of the scale until it is fully restored to the β‐lattice within different time intervals (5, 15, 30, and 60 s), which is recovered entirely involving a certain recrystallization specification, as indicated in Figure 4k–n, schematize the recovery process accordingly in Figure 4o.

**Figure 5:**
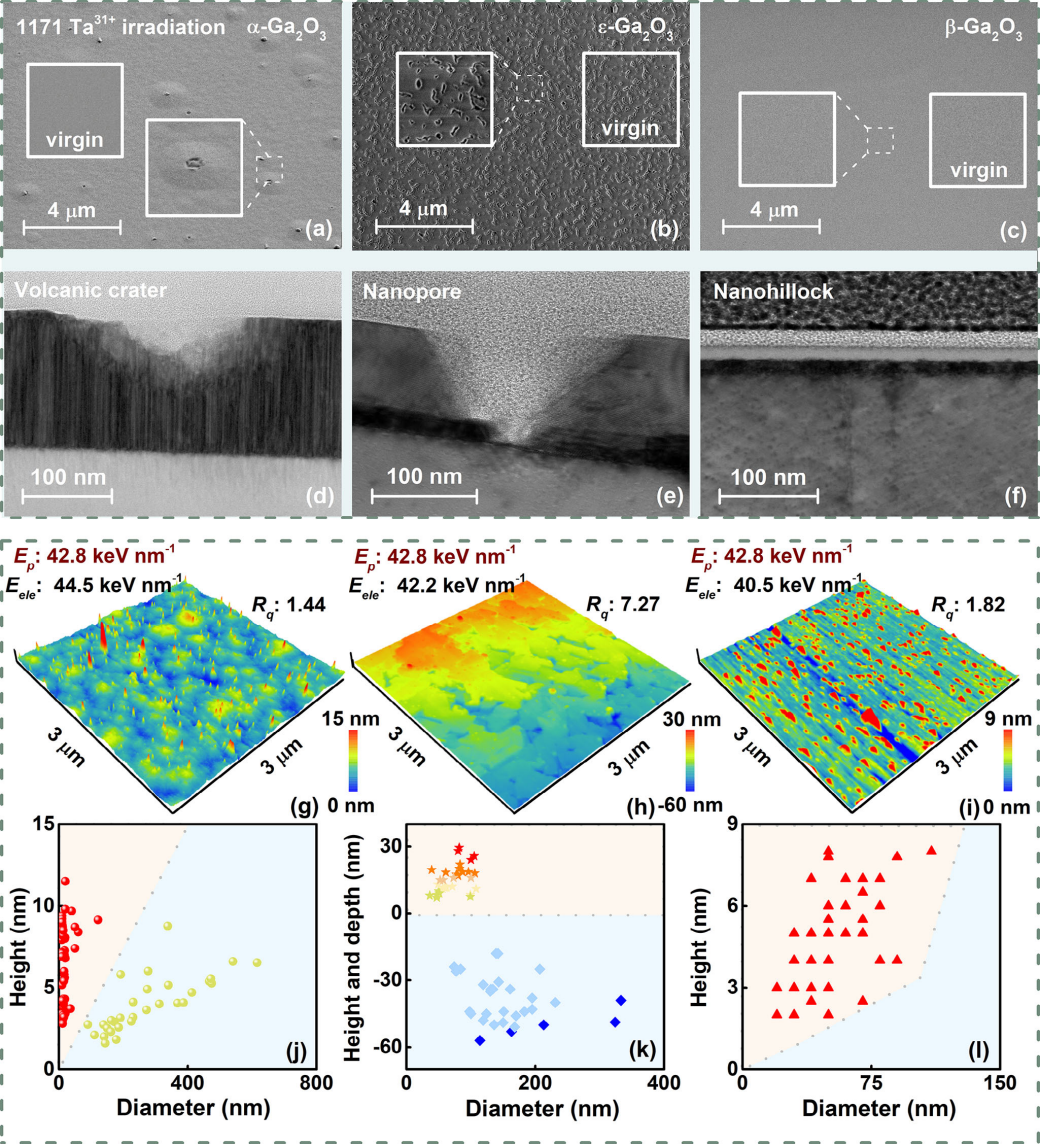
Contrasting surface morphology distributions driven by electronic excitation. (a–f) SEM images of the upper surface combined with cross‐section reflecting the morphological distribution of micro‐ and nanostructures. (g–l) Surface nanostructures in α, ε, and β‐Ga_2_O_3_. The surface morphology undergoes a transition from crater to nanopores in α and ε‐Ga_2_O_3_, while nanohillocks form in response to electronic excitation in β‐Ga_2_O_3_.

Further investigation into the evolution of surface micro‐ and nanostructures underlying irradiation‐induced electronic excitation effects, as well as the technical applicability of irradiation in nanosystems engineering was conducted. The *E_ele_* transfer from incident ions to the lattice via *e‐ph* interactions induces a localized temperature rise at lattice sites, which serves as the primary driving mechanism for molten protrusion from the surface layer. Through comprehensive SEM scanning and HRTEM analysis conducted on both large‐scale and cross‐sectional perspectives (**Figure** **5**a–f), crater‐like morphologies, nanopores, and nanohillocks have been identified in α, ε, and β‐Ga_2_O_3_, with unirradiated region inserted for comparison.

By employing AFM for nanoscale surface characterization, combined with a rigorous statistical distribution analysis, it is revealed that the three distinct morphological features are fundamentally dictated by the unique oblique rhombohedral, hexagonal, and monoclinic structural transition mechanisms inherent to each polymorphic phase, as follows: (i) under the co‐action of *E_p_* ≈42.8 keV nm^−1^ and *E_ele_* ≈44.5 keV nm^−1^ (highly charged state Ta^31+^ contributes consistent potential energy as a complementary effect on multiphase surfaces), similar to Ref. [68] a series of granular crater‐like structures protruded from the surface in α‐Ga_2_O_3_. These, including distributions up to ≈12 nm in height within ≈100 nm diameter and ≈5 nm in height in the 200–600 nm diameter range, with the surface *R_q_* of 1.44 nm (Figure 5g,j), are attributed to thermodynamic melt‐induced expansion followed by sublimation tendency to occur in domain boundaries. (ii) under *E_p_* ≈42.8 keV nm^−1^ and *E_ele_* ≈42.2 keV, the intrinsic nanopores in ε‐Ga₂O₃ enlarge to 100–300 nm. They evolve to larger structures extending from ≈60 nm below the surface to ≈30 nm above, raising the surface roughness (*R_q_*) to 7.27 nm (Figure 5h–k). The pore size and anisotropic step‐terrace arrays (step height ≈ 8 nm, matching C/2 periodicity) arise from by the competition between APBs and TDBs, with APBs prevailing.^[^ ^69^ ^]^ (iii) under the co‐action of *E_p_* ≈42.8 keV nm^−1^ and *E_ele_* ≈40.5 keV nm^−1^, small‐sized nanohillocks, accompanied by a diameter of ≈75 nm and height ranges of 2–9 nm with a surface *R_q_* of 1.82 nm in β‐Ga_2_O_3_, are attributed to microregion expansion within the internal latent track with the relatively uniform overall distribution (Figure 5i,l). Responding to intense and approximately electronic excitation, the β‐phase consists of GaO₆ octahedral co‐prismatic connections to form a layered structure, with tightly arranged atoms, high symmetry (C2/m space group), and strong Ga─O bonding (high covalency). This results in an overall structure that is not prone to collapse, and less change in the surface topography, while the α‐phase and ε‐phase are opposite, with a loosely bonded structure (some of the Ga atoms are in tetrahedral sites) corresponding to a lower atomic bonding energy, and the surface localization tends to reconfigure.^[^ ^70^ ^]^

Additionally, in the pristine samples, the intrinsic boundaries and defect‐rich regions of domain structures act as preferential sites for energy deposition, proven in Ref. [71], governing the spatial distribution of evolutionary structure, thereby localized melting, recrystallization, and thermal sublimation drive the significant interfacial inhomogeneous structure in α‐ and ε‐Ga₂O₃ than in stable β‐Ga₂O₃. Subsequently, during the process of irradiation‐induced mutual transformation between α‐ and ε‐Ga₂O₃, the propagation of TDBs disrupts the continuity of lattice planes, creating terraced surface morphologies. The steps nucleate at TDB intersections due to the minimization of interfacial strain, as the misalignment between adjacent domains necessitates discrete height adjustments to accommodate the phase‐shifted regions. Therefore, dominant boundaries of TDBs in ε‐Ga₂O₃, driven by its layered stacking structure and anisotropic surface energy, induce step‐like surfaces by disrupting lattice continuity and promoting step nucleation at phase‐shifted domains compared to TDBs in α‐Ga₂O₃, promoting further investigation of crystallographic transformation dynamics.

**Figure 6:**
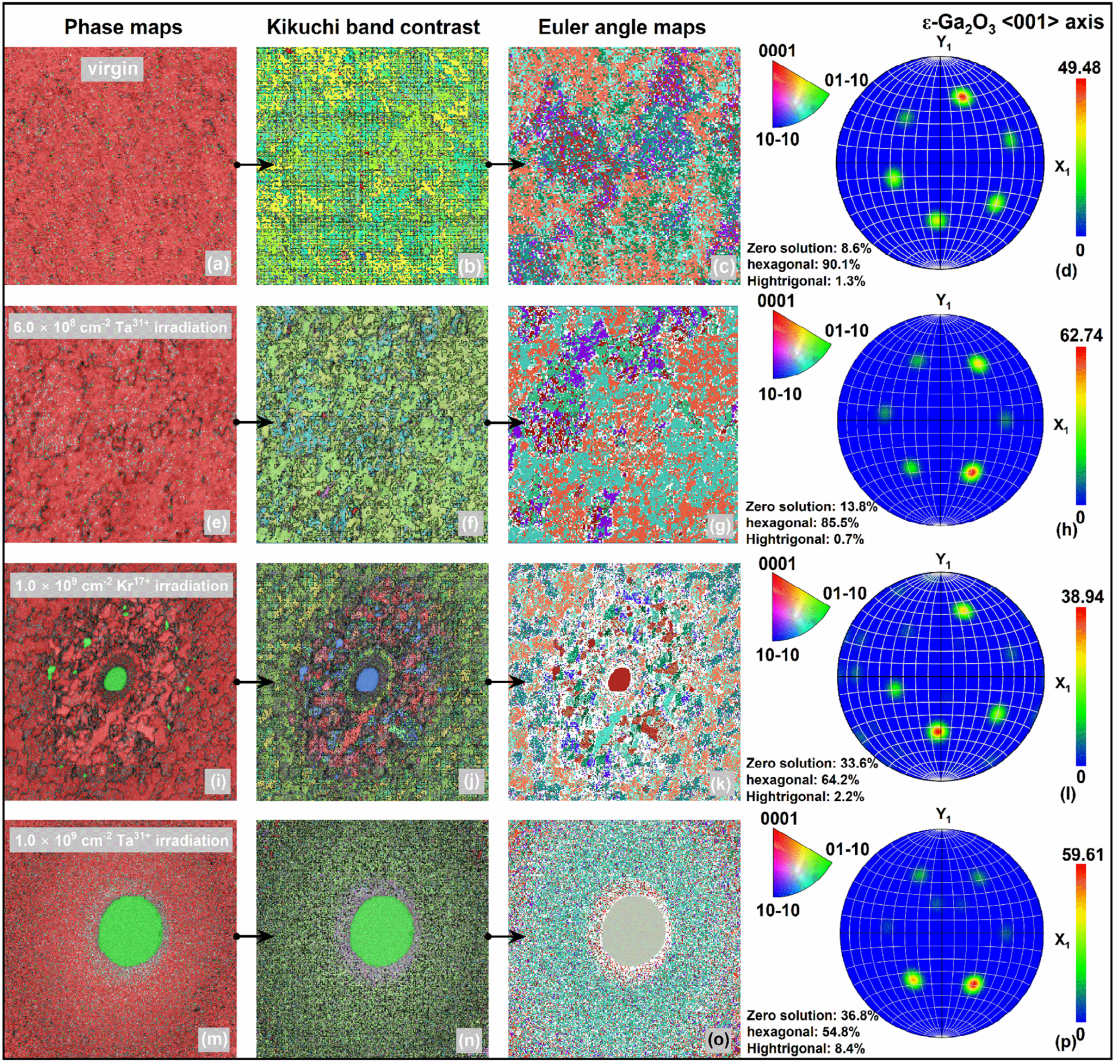
Comprehensive EBSD mapping performed across the majority of the specimen gauge section (ε‐Ga₂O₃ phase), with crystallographic orientation data presented as inverse pole figures (IPFs), normalized to a standardized reference frame. Coordinate deformation and morphology evolution of adjacent grains subjected to different irradiation conditions (Kr^17+^ and Ta^31+^). (a, e, i, m) Backscatter Channeling (BC) + Phase maps (PH), (b, f, j, n) Electron Channeling (EC) + inverse pole figure (IPF) + grain boundary (GB) maps, (c, g, k, o) Euler angle maps. (d, h, l, p) <001> pole figures for the x‐y plane.

The EBSD analysis of ε‐Ga₂O₃ nanoparticle crystals under different conditions provides insights into the phase distribution, crystallographic orientation, and microstructural changes connected to the internal domain structure induced by irradiation. In the virgin ε‐Ga₂O₃ (**Figure** **6**a–d), the predominant hexagonal phase (90.1%), low zero solution rate (8.6%, probability of failing to find a valid crystal orientation), and high‐trigonal phase (1.3%) indicate a well‐defined crystalline structure with minimal defects. The Kikuchi band contrast and Euler angle maps reveal a uniform grain orientation, suggesting a coherent domain structure with minimal internal strain or domain boundaries.

Upon 6.0 × 10^8^ cm^2^ Ta^31+^ irradiation (Figure 6e–h), the reduction in the hexagonal phase to 85.5%, coupled with an increase in the zero solution rate to 13.8%, indicates the introduction of structural defects and potential fragmentation of domains, likely due to irradiation‐induced lattice distortions. The minimal presence of the high‐trigonal phase (0.7%) suggests that phase transformation within the domains is limited, implying that the observed changes are primarily driven by defect accumulation rather than extensive phase transition. This behavior underscores the role of irradiation in disrupting the crystalline integrity and domain coherence of ε‐Ga₂O₃, while preserving the majority of the hexagonal framework with localized structural modifications. Subsequently, responding to 1.0 × 10^9^ cm^2^ Kr^17+^ irradiation (Figure 6i–l), the proportion of the hexagonal phase decreases to 64.2%, accompanied by a significant rise in the zero solution rate to 33.6%, indicative of substantial lattice distortion and the formation of new domain boundaries. Concurrently, the increase in the high‐trigonal phase to 2.2% suggests localized phase transformations within the domains, which may contribute to domain wall pinning and an elevation in internal strain. Similarly, for 1.0 × 10^9^ cm^2^ Ta^31+^ irradiation (Figure 6m–p), the reduction in the hexagonal phase to 54.8% and the increase in the high‐trigonal phase to 6.4% indicates significant phase transformation and domain reconfiguration, accompanied by a high zero solution rate of 36.8%, highlighting extensive lattice distortion and defect formation.

These structural changes disrupt the coherence of the internal domain structure, degrading crystallographic integrity and domain stability, and underscore the profound impact of irradiation on the phase evolution and microstructural dynamics. The Euler angle maps demonstrate significant randomization in grain orientation distribution in irradiated samples, contrasting with the uniform orientation in the virgin sample, which correlates with the increased zero solutions and phase transformations, indicative of irradiation‐induced domain reorientation and new domain boundary formation. Furthermore, the diminished Kikuchi band contrast in irradiated samples substantiates the introduction of lattice defects and distortions, likely impairing domain wall mobility and destabilizing the overall domain structure.

Combined with experimental observations, as evidenced by HRTEM analysis (see Figure S1e–h for details, Supporting Information, Figure 3n,o), substantial structural modifications in ε‐Ga₂O₃ are manifested through domain fragmentation and metastable phase boundary formation. Irradiation‐induced APBs in ε‐Ga₂O₃ stem from dislocation‐mediated lattice partial phase transitions, which simultaneously generate high‐trigonal symmetry domains through strain‐driven bond reconfiguration. These transitions also cause EBSD pattern degradation (zero solution rates) due to TDBs‐induced subgrain misorientation and symmetry mismatch from the original hexagonal matrix. Therefore, taking ε‐Ga₂O₃ with a multi‐domain structure characterized by three distinct crystallographic rotational axes as an example, the radiation‐sensitive nature of Ga₂O₃ domain architecture underscores the critical necessity for controlled irradiation conditions to maintain structural integrity and preserve domain‐engineered functionalities in advanced device applications. Clarifying this behavior establishes a fundamental theoretical framework for understanding irradiation‐induced phenomena and advancing the design of high‐performance Ga₂O₃‐based devices.

### 2.4 Correlation of Electronic Structure Configurations with Spectroscopic Features

**Figure 7:**
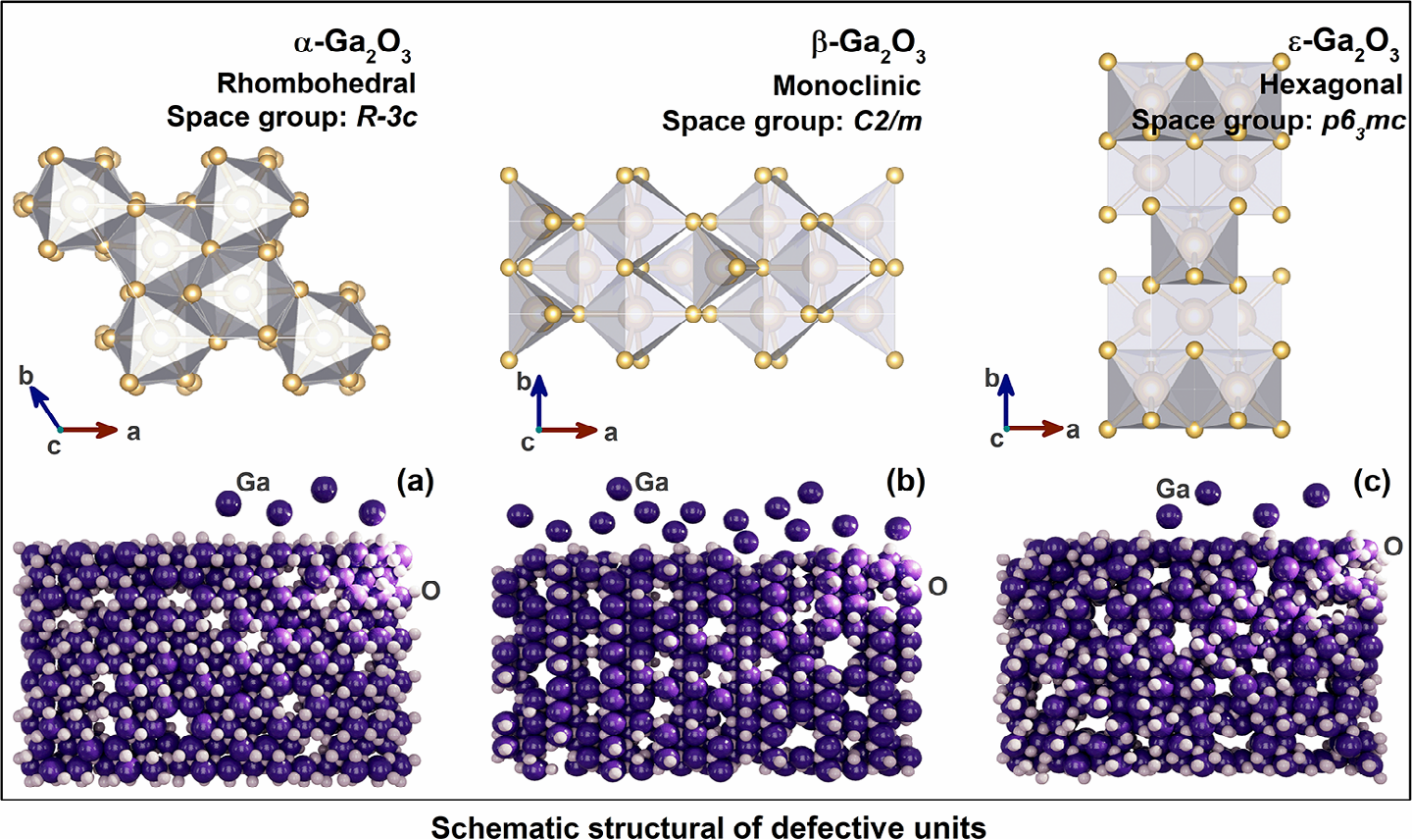
Unit cell structures and dominant defect types of α, β, and ε‐Ga_2_O_3_. (a–c) Structural unit cells of three crystal‐orientated α, β, and ε‐Ga_2_O_3_.

**Figure 8:**
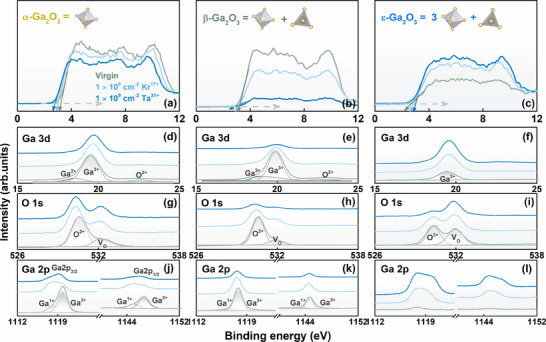
Assessment of electronic valence band structure and defect state evolution. (a–c) XPS valence‐band spectra, (d–l) the Ga 3d, O 1s, and Ga 2p, characteristic peaks determined by Gaussian fitting from experimental components of the α, β, and ε‐Ga_2_O_3_.

Associating to unique crystallographic features of α‐Ga₂O₃ (rhombohedral, *R‐3c*), β‐Ga₂O₃ (monoclinic, *C2/m*), and ε‐Ga₂O₃ (hexagonal, *p6₃mc*), **Figure** **7**a–c schematically illustrate the internal atomic defects. The distinct valence band shifts observed in α‐Ga₂O₃ and β‐Ga₂O₃ (downward) versus ε‐Ga₂O₃ (upward) post‐irradiation reveal phase‐dependent electronic restructuring under electronic excitation. For α‐ and β‐phases, the lowered valence band maximum (VBM) implies radiation‐induced defect states near the band edge or enhanced hybridization of oxygen 2p orbitals due to lattice distortion, which may correlate with vacancy‐mediated hole trapping or metastable structural rearrangements (**Figure** **8**a,b). Conversely, the elevated VBM in ε‐Ga₂O₃ likely reflects gallium vacancy V_Ga_, oxygen vacancy V_O_, and antisite defects electronic configurations in coordination environments and the charge compensation effect of dominated type defects (Figure 8c), with the basic structural units in the illustrations.^[^ ^72^, ^73^, ^74^, ^75^ ^]^

Irradiation may have facilitated overcoming the phase transition energy barrier through localized energy depositions, thereby promoting the preferential formation of the substable ε‐phase in the irradiated region. This alteration modifies the coordination sites and bond lengths associated with the Ga^3^⁺ component initially present in α‐ and β‐Ga_2_O_3_, transitioning from hexa‐coordination to tetra‐coordination, while exhibiting an opposing trend for ε‐Ga₂O₃ (Figure 8d–f). The α‐Ga_2_O_3_ phase exhibits a closely packed hexagonal structure with stable oxygen coordination, resulting in a relatively high formation energy of V_O_ under irradiation and, consequently, limited V_O_ generation (Figure 8g). Despite the irradiation preferential disrupting the tetrahedrally coordinated Ga─O bonds in the β‐Ga₂O₃ phase, which featured two distinct Ga sites (tetrahedrally and octahedrally coordinated), the overall vacancy generation efficiency remains lower than that of the ε‐phase (as depicted in Figure 8h).

The ε‐Ga_2_O_3_ phase demonstrates high local coordination flexibility, facilitating oxygen atom escape under irradiation and significantly increasing V_O_ concentration, with corresponding V_O_ acting as a nucleation site to accelerate the α/β → ε phase transition (Figure 8i). The formation of TDBs in ε‐Ga₂O₃ is evidenced to be intrinsically linked to V_O_, as V_O_‐induced lattice distortions and localized charge imbalance lower interfacial strain energy, driving coherent twinning to stabilize metastable phase configurations. The irradiation‐mediated proliferation of V_O_ could perturb the coordination geometry or oxidation state of Ga, triggering partial reduction of Ga^3^⁺ to lower‐valent species (Ga^2^⁺/Ga⁺), inducing the corresponding proportion of reduced Ga^3+^ occurs mainly in ε‐Ga_2_O_3_. The distinct Ga 2p core‐level binding energies signal attenuation in α‐/β‐Ga₂O₃ while enhancement in ε‐Ga₂O₃ correlates with phase‐specific coordination symmetry (Figure 8j–l): reduced Ga─O covalency in α/β phases lowers VBM via weakened orbital hybridization, while distorted octahedral Ga sites of ε‐phase enhance Ga 2p intensity through localized charge accumulation, simultaneously elevating VBM via strengthened Ga 4s‐O 2p antibonding interactions (Figure 8a–c).

**Figure 9:**
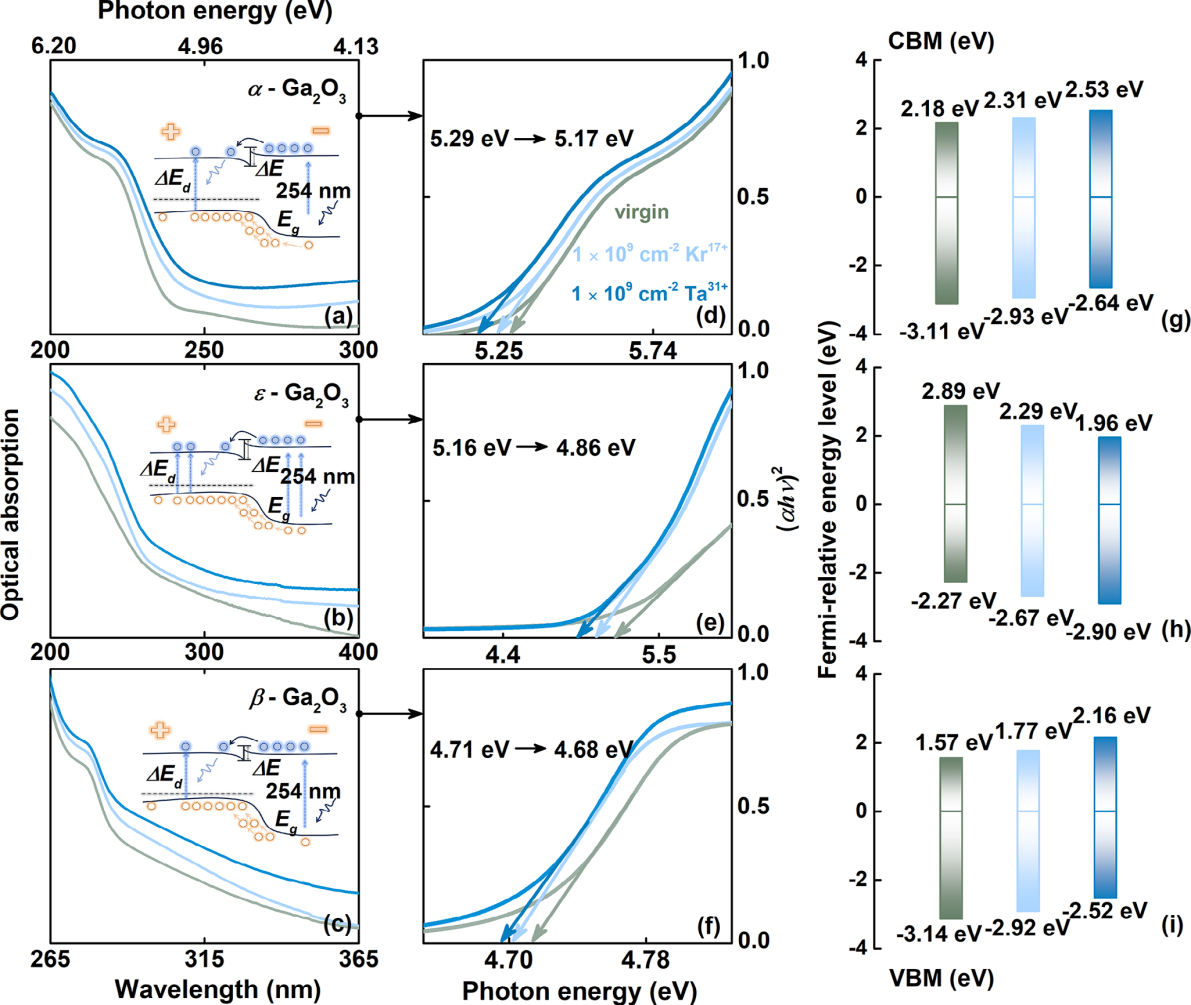
Bandgap modulation and fluorescence yield evolution driven in electronic structures. (a–f) Optical absorption spectra as a function of irradiation conditions (1.0 × 10^9^ cm^−2^ Kr^17+^ and 1.0 × 10^9^ cm^−2^ Ta^31+^), with the schematic electronic structure of α, β, and ε‐Ga_2_O_3_, exhibiting the Ga 4s derived CB and O 2p derived VB, and degenerately by electronic excitation, were displaced the Fermi level (*E_F_*) above CB minimum, making the system metallic. (g–i) VBM and CBM energy alignments of varying irradiation conditions.

The absorption spectra, bandgap narrowing, and displacement of the Fermi energy level of α‐, β‐, ε‐Ga₂O₃ polymorphs are systematically compared, accompanied by the additional fluorescence yields driven in electronic structures, under varying irradiation conditions. Schematically in the illustration, by utilizing the Tauc equation and plotting the graph of *(αhν)^2^* (*α*, absorption coefficient) versus *hν* (incident photon energy), similar to Ref. [76, 77], the enhanced light absorption in the visible band infers a gradual narrowing of the intrinsic bandgap (5.29 eV → 5.17 eV for α‐Ga_2_O_3_, 5.16 eV → 4.86 eV for β‐Ga_2_O_3_, 4.71 eV → 4.68 eV for ε‐Ga_2_O_3_, **Figure** **9**a–f), which are attributed to the additional internal defect states affecting the electronic structure. Driven by irradiation‐induced V_O_ and gallium interstitial Ga_i_ acting as shallow donors, an upward shift of VBM approximately 0.4–0.6 eV appears in α‐ and β‐phase Ga₂O₃, accompanied by an upward Fermi level migration toward CBM with enhanced electron concentration (Figure 9g,h), triggering n‐type conductance trends. Conversely, an inverse trend with a 0.6 eV downward shift of VBM and Fermi level pinning below the intrinsic position is attributed to V_O_ and V_Ga_ complexation with antisite defects of ε‐Ga₂O₃ (Figure 9i).

**Figure 10:**
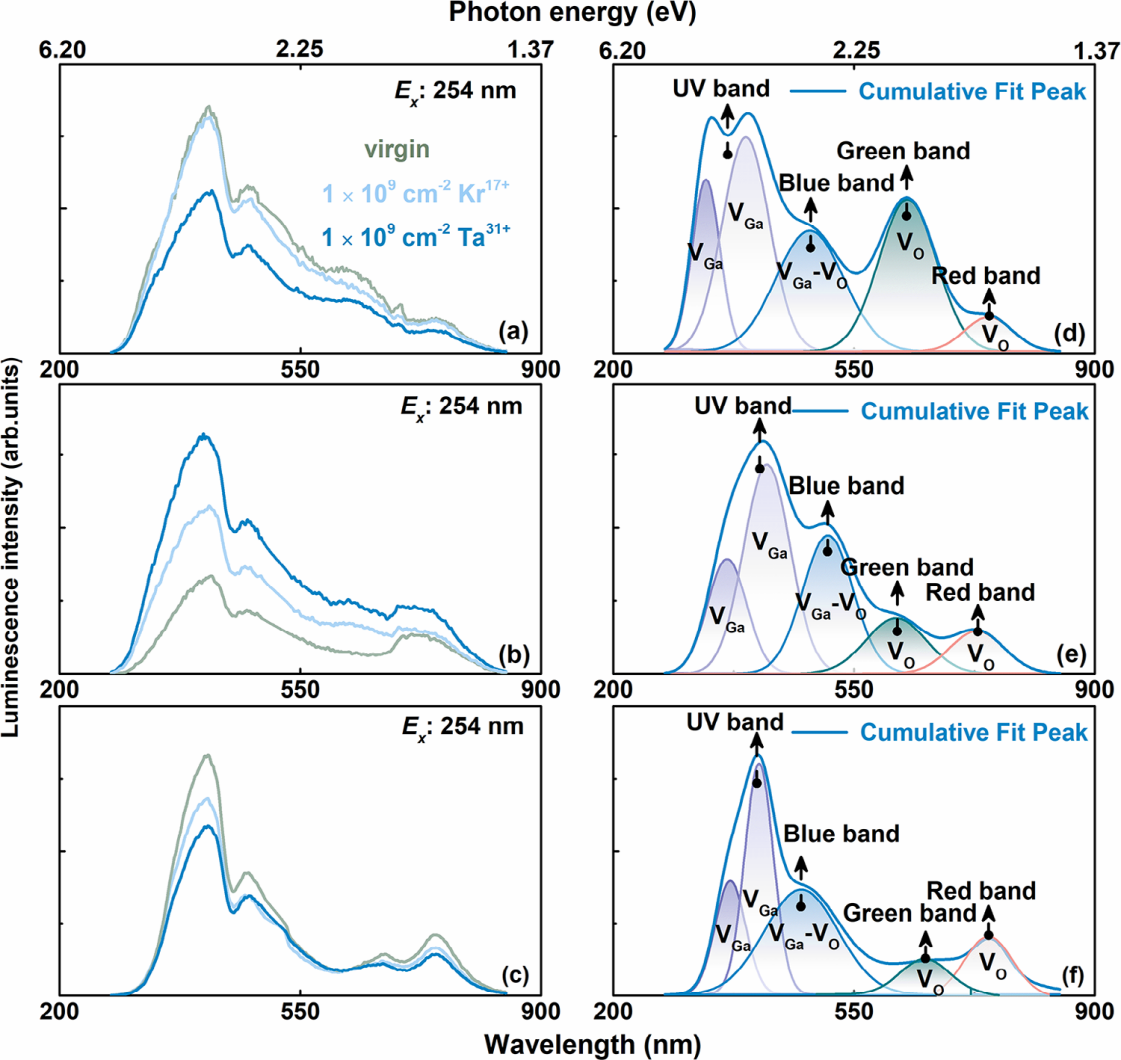
Dominant defect mechanism of induced photoluminescence response. (a‐c) photoluminescence, and (d‐f) the corresponding distribution of defect components under 1.0 × 10^9^ Ta^31+^ irradiation.

Subsequently, focusing on the distinct fluorescence at the excitation wavelength of ≈254 nm responses of Ga₂O₃ polymorphs, the kinetic evolution behaviors of internal defects associated with VBM distributions are verified, as indicated in **Figure** **10**a–f. The non‐radiative recombination centers (V_O_ and Ga_i_) suppress visible‐range photoluminescence through phonon‐mediated dissipation of excited carriers, consistent with relatively slight bandgap reductions (*ΔE* = 0.12 and 0.03 eV, respectively) that are insufficient to compensate for defect‐mediated losses in α‐ and β‐Ga₂O₃. Conversely, a significant bandgap narrowing (*ΔE* = 0.30 eV) extracted from the absorption edge in ε‐Ga₂O₃ facilitated radiative recombination efficiency, realizing the modulation of visible light emission in the sub‐wavelength band.^[^ ^78^ ^]^ Therefore, the irradiation‐induced mutual transformations of Ga_2_O_3_ phases enable effective optical regulation through defect‐configured dominance of recombination and non‐recombination responses across spectral bands.

The PL spectral deconvolution of irradiated Ga₂O₃ crystals demonstrates the irradiation‐dependent evolution of defect‐related emission bands (Figure 10a–c), and corresponding systematic analysis identifies four characteristic emission features with distinct radiation response mechanisms and underlying defects distributions,^[^ ^79^ ^]^ as follows (Figure 10d–f): (i) UV band (250 nm ~ 400 nm, 3.10 ‐ 4.96 eV), the near‐band‐edge (NBE) transition exhibits a radiation‐softened bandgap (*ΔE_g_*) due to lattice strain accumulation and shallow defect state formation, while its emission intensity is significantly suppressed (>80% attenuation at 325 nm), likely resulting from irradiation‐enhanced defect‐mediated non‐radiative recombination through newly generated mid‐gap states. (ii) Blue band (~ 488 nm, 2.54 eV), attributed to V_Ga_ or Ga─O divacancies (V_Ga─O_), irradiation‐induced lattice displacement enhances the concentration of these shallow defects. (iii) Green band (~ 550 nm, 2.25 eV), dominated by mid‐level V_O_, irradiation substantially increases V_O_ density (evidenced by the elevated V_O_ parameter), aligning with the non‐radiative recombination center. (iv) Red band (700–900 nm, 1.77–1.38 eV), likely associated with complex defect clusters (e.g., V_O_‐O_i_ complexes), irradiation‐induced lattice relaxation in metastable phases suppresses defect aggregation, resulting in diminished red emission.

To facilitate comparisons, the essential features of α‐, β‐, ε‐Ga_2_O_3_ arising from the electronic band structure are summarized in **Table** **2**, including intrinsic structure, defect state composition, and fluorescence bands measured from experimental characterization. These distinct phase‐dependent responses highlight the pivotal role of polymorph selection in engineering defect landscapes for irradiation‐modulated optoelectronic applications, and further elucidating the underlying mechanisms for other customized designs.^[^ ^80^, ^81^ ^]^

**Table 2:** Basic features of α‐, β‐, and ε‐Ga_2_O_3_ arising from the electronic structure.

Polymorph	Bandgap	System	Space group	Lattice parameters [Å]	Description or value	Feature
*α*‐Ga_2_O_3_	5.29 → 5.17 eV	Rhombohedral	R3*c*	a = 4.9825	Hybrydized Ga‐s and O‐p states	CBM/VBM
				b = 13.433	O 2p	
*ε*‐Ga_2_O_3_	5.16 → 4.86 eV	Hexagonal	P6_3_mc	a = 2.9036	V_Ga_	Visible light band: blue purple region
				a = 9.2554	V_Ga_‐V_O_	
*β*‐Ga_2_O_3_	4.71 → 4.68 eV	Monoclinic	*C*2*/m*	a = 12.214	V_O_	Visible light band: red green region
				b = 3.0371		
				c = 5.7981		

## 3 Conclusion and Future Directions

The atomic‐scale mechanisms driving polymorph‐dependent structural transformations and the associated spectral evolution in α‐, β‐, and ε‐Ga₂O₃ under intense electronic excitation have been systematically investigated, demonstrating the critical influence of specific crystallographic configurations under extreme irradiation conditions. Driven by differentiated thermodynamic excitations, the phase‐dependent energy deposition response (*E_m_*: 0.22–0.34 eV atom^−1^), associated with intrinsic ionization processes, is quantitatively shown to dominate and govern the different structural evolution pathways.

Specifically, the metastable phase interconversion via domain fragmentation and antiphase boundary proliferation emerged and competitive effects between APBs and TDBs govern the eventual phase configurations between α/ε‐Ga₂O₃. Localized β→κ/δ transitions mediated by defect clustering, driven by lattice distortion‐induced stress field, separately existed in the surface and latent track regions of β‐Ga₂O₃, reflecting the behavior of interdependent feature structures. Returning to the underlying phase recovery mechanism, the metastable κ‐phase, characterized by a corundum‐like structure and reduced thermodynamic stability, undergoes defect‐induced phase transformation. This is facilitated via localized electronic excitations that lower atomic rearrangement energy barriers, where structural defects act as nucleation sites for β‐phase reconfiguration. In α‐ and ε‐Ga_2_O_3_, domain‐boundary‐mediated energy deposition drives defect accumulation alongside phase‐configurational dominance and non‐recoverable structural properties. Responding to electronic excitation combined with potential energy deposition, β‐Ga₂O₃ consists of GaO₆ octahedral framework (C2/m symmetry) and high Ga─O covalency resulting in nanohillocks. The α/ε‐Ga₂O₃ exhibits crater‐like nanopores driven by structural looseness (tetrahedral Ga sites) and reduced atomic bonding energy, revealing phase‐dependent surface dynamics governed by atomic packing density and bonding anisotropy. These phase‐specific transformations and interaction of dominant defects play a critical role in bandgap narrowing and dictate radiative and non‐radiative recombination pathways. Ultimately, opposing trends were observed in visible‐range photoluminescence yield between α‐/β‐Ga₂O₃ and ε‐Ga₂O₃, establishing the atomistic‐to‐macroscopic performance correlation framework that fundamentally bridges intrinsic defect mechanisms with spectral behaviors.

Building on the current findings, the β‐phase is positioned as the optimal candidate for next‐generation power electronics due to its exceptional radiation recovery dynamics. Defect‐mediated optical tunability in α‐/ε‐phase variants enables irradiation‐responsive sensing applications, driving future investigations toward multiscale defect dynamics modeling, synergistically integrated with irradiation parameter optimization, to establish a transformative defect‐engineering paradigm in Ga_2_O_3_ systems.

## 4 Experimental Section

### Sample Preparation and Ion Irradiation Process

The α‐ and ε‐Ga_2_O_3_ crystals were grown directly on a 2‐inch c‐plane single‐polished sapphire substrate using a self‐developed hot‐wall vertical structure mist‐chemical vapor deposition (mist‐CVD) system. Gallium acetylacetonate (Ga(acac)_3_) was dissolved in deionized water to form a 0.05 M aqueous solution, and 1% VOL hydrochloric acid (36.8%) was added dropwise to the solution to ensure that the Ga(acac)_3_ was completely dissolved. In this solution, Ga(acac)_3_ served as the gallium precursor, while H_2_O acted as the oxygen source. The aqueous gallium acetylacetonate solution was atomized to form micron‐sized droplets using an ultrasonic atomization device with a frequency of 1.7 MHz, and the atomized gallium precursor was transported to the reaction chamber via a carrier gas. High‐purity argon (99.999%) served as the carrier gas, while high‐purity oxygen (99.999%) acted as the diluting gas, with flow rates maintained at 1600 sccm for α‐Ga_2_O_3_, 2000 sccm for ε‐Ga_2_O_3_ and 200 sccm for α‐Ga_2_O_3_, 600 sccm for ε‐Ga_2_O_3_, respectively. The entire growth process occurred under atmospheric pressure conditions, maintaining a consistent growth temperature of 500 °C in α‐Ga_2_O_3_ and 640 °C in ε‐Ga_2_O_3_ for a duration of 2 h. The β‐Ga₂O₃ crystals growth was performed using the innovative edge‐defined film‐fed growth (EFG) method equipped with radio frequency induction heating. The raw Ga_2_O_3_ with a purity of 99.999% was pressed into flakes. The pressed flakes were then sintered in an air atmosphere at 1200 °C for 6 h to remove adsorbed water. The prepared material was loaded into an iridium crucible equipped with an iridium die. After the raw material was completely melted, a seed crystal was used to pull the crystal at a rate of 10 mm/h. The entire growth process was carried out in an atmosphere of 1% O_2_, 70% CO_2,_ and 29% N_2_, and the entire crystal growth process was conducted at atmospheric pressure. After growth, the crystal was slowly cooled to room temperature at a rate of 20–30 °C per hour. Followed by a surface polishing, optically polished α‐, β‐, and ε‐Ga_2_O_3_ crystals with an ⟨100⟩ orientation, featuring rhombohedral, monoclinic, and hexagonal structure domains, were cut into 10 × 10 × 1 mm^3^ employed in this research.

Irradiation experiments were conducted using 430.0 MeV ^84^Kr^17+^ and 1171.0 MeV ^181^Ta^31+^ at 300 K utilizing the Space Environment Simulation and Research Infrastructure (SESRI), Institute of Modern Physics (IMP), Harbin Institute of Technology (HIT). The fluences varied from 6.0 × 10^8^ to 1.0 × 10^9^ cm^−2^, based on a relatively low ion flux ≈4.5 × 10^4^ cm^−2^ s^−1^ maintained throughout the irradiation process, ensuring uniformity and stability of irradiation.

### Characterization of Micro/Nanostructures and Photoelectric Properties

For microstructural characterizations, High‐resolution X‐ray diffraction (HRXRD) measurements were performed using a Rigaku Smartlab diffractometer with a parallel and monochromatic X‐ray beam with a wavelength of 1.54 Å. Reciprocal space maps (RSM) were also recorded for the symmetric ⟨1200⟩ lattice planes of the samples both pristine and irradiated with Kr^17+^ and Ta^31+^, determining the in‐plane and out‐plane lattice strain. X‐ray photoelectron spectroscopy (XPS) measurements were performed using an Escalab 250Xi multifunctional imaging electron spectrometer (Thermo Scientific, USA), equipped with a hemispherical electron energy analyzer. Monochromatic Al Ka X‐rays were utilized as the incident radiation source. Narrow high‐resolution scans were acquired with a step size of 0.05 eV. Raman spectroscopy measurements of damage cross‐sections were recorded using a HORIBA LabRAM HR Evolution Confocal Raman spectrometer equipped with a 532 nm Nd‐YAG laser for excitation. Cross‐sectional α‐, β‐, and ε‐Ga₂O₃ samples, prepared via a focused ion beam (FIB) system, were characterized via high‐resolution transmission electron microscopy (HRTEM) using an FEI Tecnai G2 F20 transmission electron microscope operated at 200 kV and high‐angle annular dark‐field scanning transmission electron microscopy (HAADF‐STEM) using an aberration‐corrected scanning transmission electron microscope (Spectra 300, Thermo Fisher) operated at 300 kV. The surface morphologies were characterized using a field emission scanning electron microscope (SEM, JSM‐7610F) and the atomic force microscopy (AFM) module of an SPM‐9700HT scanning probe microscope, employing a silicon tip (resonance frequency = 70 kHz, force constant = 0.5 N m^−1^) in tapping mode. The AFM measurements were conducted at room temperature in ambient air, with the manufacturer‐reported radius of curvature of the tip being less than ≈10 nm. For photoelectric measurements, absorbance regulations were determined using an Agilent Cary 5000 UV–vis spectrophotometer within the UV–vis range. Photoluminescence (PL) spectra were acquired using an Edinburgh FLS920 all‐functional fluorescence spectrometer using xenon lamps with wavelengths of ≈254 nm as excitation light sources.

### Comparison of Critical Thermodynamics and Structural Parameters

The distribution profiles of electronic energy loss (*E_ele_*) in α‐, β‐, ε‐Ga_2_O_3_ crystals induced by 430 MeV Kr^17+^ and 1171 MeV Ta^31+^ irradiations were determined by the stopping and range of ions in matter (SRIM) 2013 simulation code,^[^ ^59^, ^60^ ^]^ with the densities of 6.44, 5.90, and 6.10 g cm^−3^, utilizing the detailed calculation with full damage cascades.^[^ ^82^, ^83^ ^]^ As summarized in **Table** **3**,^[^ ^84^, ^85^, ^86^, ^87^, ^88^ ^]^ based on the thermodynamic parameters (specific heat coefficient *c_a_* and thermal conductivity *K_a_*) at varying temperatures, numerical simulations were conducted to investigate the irradiation energy exchange, diffusion processes, and temperature evolution in the electronic and atomic subsystems. These simulations were performed under different ion velocities corresponding to *E_ele_*, utilizing the inelastic thermal spike (i‐TS) model.^[^ ^35^, ^64^, ^89^, ^90^, ^91^ ^]^

**Table 3:** The i‐TS model calculation of thermal properties in α‐, β‐, and ε‐Ga_2_O_3_ systems.

Comparison of thermal properties of *α‐, β‐, ε‐*Ga_2_O_3_ systems along <100> orientations (*RT* ≈300 K)				
Sample	Orientation	Specific heat capacity	Thermal conductivity	Thermal expansion
		[J kg^−1^ K^−1^]	[W m^−1^ K^−1^]	[10^−6^ K^−1^]
Ga_2_O_3_	α ⟨100⟩	480	18	5.2
	β ⟨100⟩	470	21	4.3
	ε ⟨100⟩	490	12	5.5

## Conflict of Interest

The authors declare no conflict of interest.

## Author Contributions

X.H. was responsible for conceptualization, formal analysis, data curation, and writing the original draft. Y.L. contributed to the investigation. Y.Li. also participated in the investigation. M.L.C. provided supervision and contributed to writing through review and editing. E.Z. offered supervision and took part in writing through review and editing. W.M. provided supervision and assisted in writing through review and editing. P.L. was involved in conceptualization, visualization, and formal analysis, contributed to writing the original draft, and secured funding for the project.

## Supporting information



Supporting Information

## Data Availability

The data that support the findings of this study are available from the corresponding author upon reasonable request.
