# Supplementary material for: Unraveling the Atomic Mechanism of the Crystalline Phase‐Dependent Structural Features and Special Spectral Design of α‐, β‐, and Ɛ‐Ga₂O₃
Source: Adv Sci (Weinh). 2025 Jun 23;12(31):e08207. doi: 10.1002/advs.202508207 (PMC12376511; doi:10.1002/advs.202508207)
Supplement: Supplementary file 1 — Supporting Information [file ADVS-12-e08207-s001.docx]

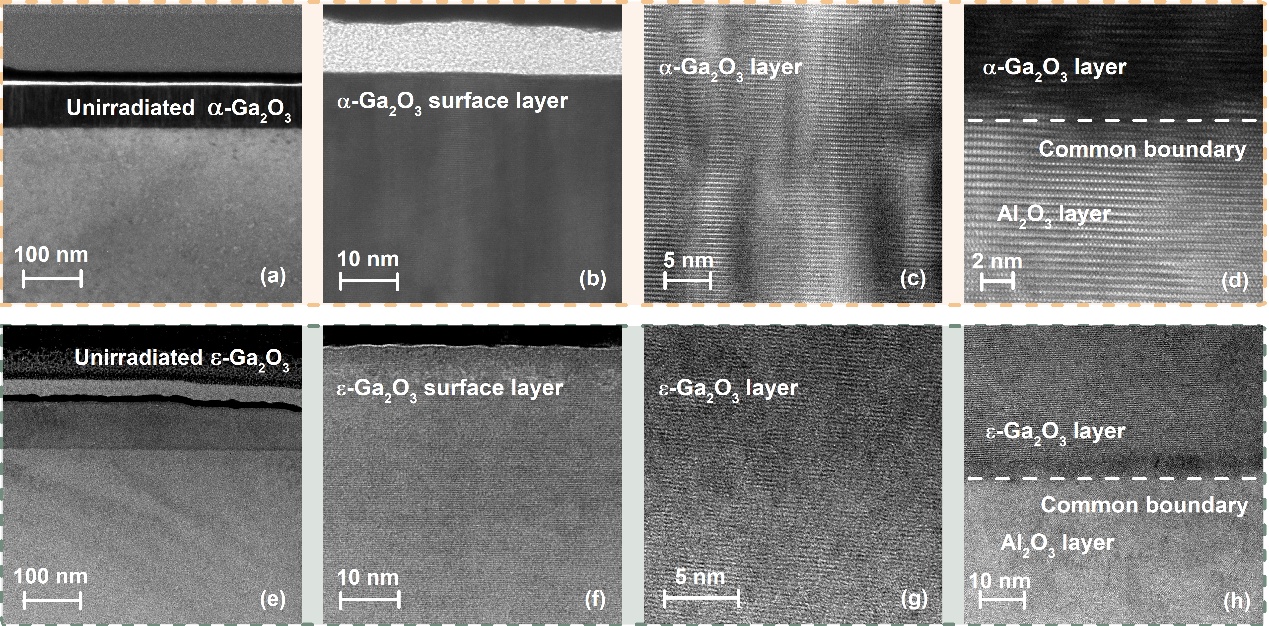


**Figure S1 The intrinsic atomic phase of the unirradiated α-Ga_2_O_3_ and ε-Ga_2_O_3_.** Cross-sectional TEM micrographs of unirradiated α-Ga_2_O_3_ and ε-Ga_2_O_3_, involving surface (a-c) α-Ga_2_O_3_, (e-g) ε-Ga_2_O_3_ and (d, h) Al_2_O_3_ substrate layer at different magnification scales.
